# Morphological and Ecological Traits Explain Variation in Desiccation Tolerance Among Bees

**DOI:** 10.1002/ece3.74355

**Published:** 2026-09-20

**Authors:** Victor H. Gonzalez, Anna Dreusicke, Meredith G. Johnson, Thomas Tscheulin, Theodora Petanidou, John M. Hranitz, John F. Barthell

**Affiliations:** ^1^ Biodiversity Institute and Department of Ecology & Evolutionary Biology University of Kansas Lawrence Kansas USA; ^2^ Department of Biology University of Iowa Iowa City Iowa USA; ^3^ Department of Biological Sciences North Dakota State University Fargo North Dakota USA; ^4^ Laboratory of Biogeography and Ecology, Department of Geography University of the Aegean, University Hill Mytilene Greece; ^5^ Department of Biology Commonwealth University of Pennsylvania Bloomsburg Pennsylvania USA; ^6^ Department of Biology University of Central Oklahoma Edmond Oklahoma USA

**Keywords:** climate change, physiological ecology, pollinator ecology, trait‐based ecology

## Abstract

Rising temperatures associated with global warming are often accompanied by shifts in precipitation patterns, increasing the risk of desiccation for insects. However, our understanding of how functional traits shape desiccation tolerance remains limited, particularly among pollinators. We assessed the desiccation tolerance of 22 bee species (13 genera, five families) from the Greek island of Lesbos, covering a range of body sizes, hairiness, nesting habitats, and social structures. We measured three desiccation tolerance metrics under standardized acute desiccating conditions (27°C and 0% RH): survival time, water loss rate (WLR), and water content at mortality (critical water content, CWC). In addition, we evaluated the phylogenetic signal of these three desiccation‐related traits and examined the influence of morphology (body mass and hair length), nesting (nest guild and nest location), and sociality on each. Survival time, WLR, and CWC varied widely among species. Larger‐bodied species exhibited lower WLR and survived longer under desiccation stress. Survival time decreased with increasing WLR and CWC. Hair length was positively associated with CWC but did not affect survival time or WLR. Ecological traits did not predict survival time, but nesting guild influenced both WLR and CWC, and sociality influenced CWC. Eusocial species nesting in large cavities exhibited higher WLR and CWC, whereas solitary species generally showed lower CWC. Phylogenetic signal was moderate (WLR) to strong (survival time and CWC) but was not statistically significant. Our results reveal significant interspecific variation in bee desiccation tolerance and demonstrate that morphological and ecological traits shape physiological responses to water stress. These results highlight trait‐based mechanisms underlying desiccation tolerance and suggest that functional trait variation may influence pollinator vulnerability to increasing drought and climatic variability.

## Graphical abstract

We assessed the desiccation tolerance in 22 bee species from the Greek island of Lesbos. Larger bees lost water more slowly and survived longer, while survival declined with increasing water loss rate and water content at mortality. Morphological and ecological traits shaped physiological responses, highlighting trait‐based mechanisms underlying pollinator vulnerability to drought.

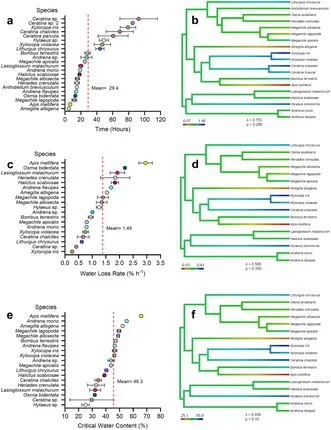

## 1 Introduction

Water balance is critical to physiological processes and plays a key role in shaping the ecology, distribution, and behavioral responses of organisms (e.g., Bujan et al. 2016; Kellermann and van Heerwaarden 2019; Sinclair et al. 2024). Traits such as cuticular permeability, critical water content thresholds, and body size can determine species' responses to desiccation stress and may become increasingly important under climate change. Rising global temperatures are often accompanied by shifts in precipitation patterns and droughts (IPCC 2021), which can increase evaporative water loss and reduce the availability of environmental water. However, most climate change studies have focused on temperature effects even though thermal and hydric stress are closely linked and that warming may intensify desiccation risk (Harvey et al. 2023). As a result, our understanding of insect desiccation tolerance and water balance traits remains limited, especially in ecologically and economically important groups such as bees (e.g., Botsch et al. 2024; Gonzalez et al. 2024).

Bees are undeniably the most important animal pollinators on the planet, and both ecosystems and global food security depend on them (Michener 2007; Klein et al. 2007). Over the past few decades, several studies have documented declines in bee abundance and diversity worldwide, driven by human‐induced stressors, including climate change, which is considered one of the leading factors (e.g., Goulson et al. 2015; Wagner 2020; Kougioumoutzis et al. 2022; Cornelisse et al. 2025). Bees consist of more than 20,000 species worldwide, varying greatly in morphology, behavior, and nesting ecology (Michener 2007; Danforth et al. 2019). These differences expose them to a wide range of microclimates and potentially shape their vulnerability to environmental stressors. For example, bees vary dramatically in body size (2–30 mm), color, hairiness, floral specialization, nesting habits (both below and above ground) and social organization (ranging from solitary to eusocial). Comparative studies linking physiological water balance traits with ecological strategies across bee species are rare. As a result, it remains unclear whether desiccation tolerance in bees is primarily driven by morphological or physiological constraints such as body size and water balance traits, or whether ecological traits, such as nesting habits and sociality, play a major role.

Existing studies indicate that bees tend to have water contents comparable to that of other insects (Gonzalez et al. 2020; Botsch et al. 2024; Johnson, Glass, et al. 2023) but also reveal significant interspecific variation in desiccation tolerance and critical water content or the percentage of water content at mortality (Burdine and McCluney 2019; da Silva et al. 2021; Gonzalez et al. 2023; Botsch et al. 2024). Critical water content (CWC) declines with increasing body mass (Glass et al. 2025), suggesting that larger bees may better withstand greater proportional body water loss before lethal dehydration. These variations have been linked to differences in foraging patterns (Gonzalez et al. 2023) or habitat choice (da Silva et al. 2021), demonstrating the ecological significance of desiccation tolerance in shaping bee survival and distribution.

Here we assess variation in desiccation tolerance across 22 species of bees from five families on the Greek island of Lesbos, a global hotspot for bee diversity (Kougioumoutzis et al. 2022). These species differ in several features, including body size, hairiness, nesting habits, and social organization. They range from small, nearly hairless, ground‐nesting social bees [ *Lasioglossum malachurum* (Kirby)] to large, hairy, wood‐nesters, solitary bees [ *Xylocopa violacea* (Linnaeus)]. We quantified three desiccation tolerance metrics: survival time (i.e., time to death) under acute desiccating conditions, water loss rate (WLR), and critical water content (CWC). These traits provide insight into the whole‐organism performance, water retention efficiency, and the threshold at which dehydration becomes lethal, respectively (Botsch et al. 2024). We expect both WLR and CWC to be inversely correlated with survival time, as individuals that lose water more rapidly and require a higher minimum water content to survive would be more susceptible to desiccation than those that lose water slowly and can tolerate lower body water levels.

We tested three main hypotheses: First, we predict that larger bees would exhibit greater desiccation tolerance due to a lower surface‐area‐to‐volume ratio than smaller bees (Kühsel et al. 2017). Second, we predict that increased body hair length will enhance desiccation tolerance, as hairiness reduces water loss in ants and other arthropods by creating a boundary layer over the cuticle (Buxton et al. 2021). Third, we predict that ecological traits would influence desiccation tolerance: bees nesting above ground are generally exposed to greater fluctuations in humidity and higher ambient temperatures than ground‐nesting bees, thus they will exhibit higher desiccation tolerance. Similarly, solitary species will exhibit higher desiccation tolerance than social species because they are unable to regulate nest humidity through behavioral or collective mechanisms (e.g., Ellis et al. 2008; Nicolson 2009; Gonzalez et al. 2022; Ostwald, da Silva, and Seltmann 2024). Finally, because physiological traits may be constrained by evolutionary history (e.g., Kellermann et al. 2018), we also assessed the phylogenetic signal of survival time under desiccating conditions, WLR, and CWC. We predict a strong phylogenetic signal in these traits, as closely related species often have similar body sizes, share nesting strategies, and levels of sociality. For example, all species of *Andrena* Fabricius (Andrenidae) are ground nesters and solitary (Michener 2007). To date, no study has systematically linked physiological water balance traits to ecological and phylogenetic factors in bees, and our study aims to start filling this knowledge gap.

## 2 Material and Methods

### 2.1 Study Sites and Bee Collections

Details of sampling locations and collecting protocols are in Gonzalez et al. (2020, 2024). We captured bees during June and July of 2024 at two sites on the Greek island of Lesbos: Achladeri, a coastal seminatural low scrub (phrygana) habitat close to Ancient Pyrrha (39°09′17.40″N, 26°16′32.74″E, 1.3 ma.s.l.), and an unmanaged pasture about 9 km northwest from Achladeri, near Skala Kallonis (39°12′36″N, 26°12′12″E, 1.0 ma.s.l.). Bees were collected at flowers between 6:00 and 11:00 h using a net and placed individually into plastic vials capped with fabric (with holes ~1 mm in diameter). We maintained specimens in a cooler with an ice pack wrapped in cloth (16°C–19°C) until fieldwork was completed. We chose species based on local abundance and variation in morphology and life‐history traits, including differences in body size and hairiness, ground‐ and wood‐nesting habits, and solitary and social behaviors (Table 1). We aimed to collect at least 20 individuals per species; however, because bees were sampled opportunistically, sample sizes differed among species depending on their relative abundance in the field. To increase species representation and sample sizes, we complemented collections from an unmanaged pasture near Agiassos (39°04′15.4″N, 26°23′32.2″E, 625 m.a.s.l.), approximately 12 km southwest of Achladeri. Most bees in our study lacked pollen loads in the corbiculae or noticeable pollen on the scopae, as they were collected either from flowers that are primarily visited for nectar (e.g., *Vitex agnus‐castus* L., Lamiaceae) or when leaving their nests, as in the case of sweat bees. Individuals carrying visible pollen loads were excluded to avoid inflating body mass measurements. We brought bees to the laboratory and, to ensure similar levels of hydration as well as nutrition, we fed them *ad libitum* for 30 min with a 50% sucrose solution before desiccation tolerance assays (see pilot assay below). We kept bees in the dark to minimize stress and frequently observed individuals feeding, but we did not record these observations. Bees were tested within 1–2 h of being collected in the field. The senior author identified specimens by comparing them with the reference bee collection of the Melissotheque of the Aegean at the University of the Aegean, Mytilene, Lesvos, Greece.

**TABLE 1:** Life‐history, morphological, and desiccation‐related traits of bee species used in desiccation tolerance assays from the Greek island of Lesbos.

Species	Life history			Morphology		Desiccation		
	NG	NL	SL	WM	HL	ST	WLR	CWC
Andrenidae								
*Andrena flavipes*	G	BG	S	97, *n* = 1	0.60, *n* = 1	14, *n* = 1	1.69, *n* = 1	46.45, *n* = 1
*Andrena morio*	G	BG	S	173, *n* = 1	1.10, *n* = 1	19, *n* = 1	0.79, *n* = 1	55.21, *n* = 1
*Andrena* sp.	G	BG	S	42.5 ± 8.50, *n* = 2	0.50, *n* = 1	27 ± 7, *n* = 2	1.05 ± 0.03, *n* = 2	43.8 ± 2.37, *n* = 2
Apidae								
*Amegilla albigena*	G	BG	S	78, *n* = 1	0.46, *n* = 1	5, *n* = 1	1.54, *n* = 1	52.27, *n* = 1
*Apis mellifera*	L	AG	E	92.8 ± 2.94, *n* = 37	1.22 ± 0.01, *n* = 37	6.49 ± 1.58, *n* = 37	2.96 ± 0.25, *n* = 37	65.85 ± 0.97, *n* = 35
*Bombus terrestris*	L	BG	E	434.1 ± 41.9, *n* = 18	1.83 ± 0.06, *n* = 18	28.44 ± 3.74, *n* = 18	0.95 ± 0.09, *n* = 18	47.04 ± 1.19, *n* = 17
*Ceratina chalcites*	H	AG	S	37.5 ± 6.35, *n* = 6	0.19 ± 0.01, *n* = 6	68.8 ± 17.3, *n* = 6	0.67 ± 0.22, *n* = 6	34.33 ± 2.44, *n* = 6
*Ceratina parvula*	H	AG	P	3.4 ± 0.001, *n* = 7	0.10 ± 0.02, *n* = 7	60.4 ± 18.9, *n* = 7	NA	NA
*Ceratina* sp.	H	AG	S	41.5 ± 4.5, *n* = 2	0.58 ± 0.38, *n* = 2	92.5 ± 23.5, *n* = 2	0.42 ± 0.01, *n* = 2	29.6 ± 16.1, *n* = 2
*Ceratina* sp. 2	H	AG	S	4, *n* = 1	0.21, *n* = 1	85, *n* = 1	NA	NA
*Xylocopa iris*	H	AG	S	227.8 ± 16.9, *n* = 11	1.11 ± 0.09, *n* = 12	79.2 ± 9.8, *n* = 12	0.27 ± 0.04, *n* = 11	46.34 ± 1.71, *n* = 9
*Xylocopa violacea*	H	AG	S	632.8 ± 44.0, *n* = 20	1.48 ± 0.03, *n* = 20	46.9 ± 10.3, *n* = 20	0.76 ± 0.11, *n* = 20	46.22 ± 1.07, *n* = 18
Colletidae								
*Hylaeus* sp.	H	B	S	19.25 ± 0.98, *n* = 8	0.06 ± 0.03, *n* = 8	48.9 ± 5.6, *n* = 8	1.25 ± 0.11, *n* = 8	25.09 ± 2.73, *n* = 8
Halictidae								
*Halictus scabiosae*	G	BG	P	60.47 ± 2.2, *n* = 19	0.64 ± 0.01, *n* = 19	18.3 ± 1.8, *n* = 19	1.83 ± 0.14, *n* = 19	38.89 ± 1.01, *n* = 19
*Lasioglossum malachurum*	G	BG	P	14.2 ± 0.86, *n* = 15	0.25 ± 0.01, *n* = 15	19.87 ± 2.56, *n* = 15	1.92 ± 0.27, *n* = 14	32.10 ± 4.12, *n* = 14
Megachilidae								
*Anthidiellum breviusculum*	A	AG	S	18, *n* = 1	0.1, *n* = 1	15, *n* = 1	NA	NA
*Heriades crenulata*	H	B	S	14.4 ± 0.93, *n* = 5	0.11 ± 0.004, *n* = 5	15.0 ± 3.21, *n* = 5	1.84 ± 0.54, *n* = 5	32.80 ± 6.18, *n* = 5
*Lithurgus chrysurus*	H	AG	S	90.5 ± 2.83, *n* = 18	0.42 ± 0.01, *n* = 18	43.89 ± 5.26, *n* = 18	0.62 ± 0.04, *n* = 17	40.35 ± 1.17, *n* = 18
*Megachile albisecta*	H	B	S	88.16 ± 6.1, *n* = 25	0.55 ± 0.02, *n* = 25	15.56 ± 2.68, *n* = 25	1.35 ± 0.20, *n* = 24	48.96 ± 1.13, *n* = 25
*Megachile apicalis*	H	B	S	40, *n* = 1	0.02, *n* = 1	25, *n* = 1	0.80, *n* = 1	43.68, *n* = 1
*Megachile lagopoda*	H	B	S	79.2 ± 3.54, *n* = 12	0.56 ± 0.02, *n* = 12	11.67 ± 3.09, *n* = 12	1.39 ± 0.20, *n* = 11	49.57 ± 1.41, *n* = 11
*Osmia bidentata*	H	B	S	28, *n* = 1	0.08, *n* = 1	13, *n* = 1	2.20, *n* = 1	31.86, *n* = 1

*Note:* Life history: Nesting guild (NG): A = aerial; G = ground‐nesters; H = hole‐nesters; L = large cavity nesters. Nest location (NL): AG = above ground; BG = below ground; B = both. Sociality (SL): E = eusocial; P = socially polymorphic; S = solitary. Morphology: WM = wet body mass (mg); HL = hair length (mm). Desiccation: ST = survival time (hours); WLR = water loss rate (%h^−1^); CWC = critical water content (%). Average values followed by standard error (±) and sample size.

Abbreviation: NA, missing data.

### 2.2 Desiccation Tolerance Assays

To assess differences in bees' ability to tolerate acute desiccation stress, we placed individual bees in 50 mL Falcon tubes (30 × 115 mm; Falcon, Corning, NY, U.S.A.) sealed with a 1 mm mesh and attached to a similar tube containing fully dehydrated Drierite desiccant (W.A. Hammond Drierite Co. Ltd., Xenia, OH). We drilled a 1.0‐cm opening in the tube lids and secured the mesh between the lids using a hot glue gun. Both tube lids were externally reinforced with duct tape and sealed with parafilm. We used a circular layer of cotton inside the lid of the tube with the desiccant to prevent small particles of Drierite from entering the tube containing the bee. We also placed a wire mesh inside the tube with the bee to prevent it from chewing the mesh and moving into the desiccant tube. The latter adjustment was necessary for species in the genera *Xylocopa*, *Lithurgus*, and *Megachile*, which during pilot assays, chewed through the regular mesh. The relative humidity inside the desiccation apparatus was 0%, as determined by an iButton data logger (DS1923 Hygrochron; Maxim Integrated, San Jose, California). The desiccation apparatus used here was initially developed for ants (Bujan et al. 2016) but has also been used with bees (Gonzalez et al. 2023, 2024). We conducted assays at room temperature (~27°C), with each bee remaining in the apparatus until death. Thus, exposure duration to desiccating conditions varied among individuals and corresponded to survival time. We monitored each bee hourly between 6:00 and 23:00 h and every 2 h overnight. At each observation, we gently tapped and rotated the tube and considered an individual dead when it exhibited no body movement in response to this stimulation. We kept bees in the dark inside a cardboard box to minimize potential physiological and behavioral stress associated with light and recorded time of death as the response variable. We measured body mass of each specimen prior to the assays. After assays concluded, we recorded body mass at death, dry body mass, and hair length, as described below.

### 2.3 Feeding Status and Desiccation Assay Protocol Refinement

The time of death following acute desiccation exposure may be influenced by the bees' feeding status. Thus, to assess the impact of this variable and refine the desiccation assay protocol outlined above, we conducted a pilot experiment using honey bees fasted for 24 h. We captured honey bees in individual plastic vials from two experimental Langstroth hives trained to forage at a feeder containing a 50% sucrose solution scented with lavender or mint. To ensure similar feeding conditions, we used a micropipette to feed bees to satiation with a 50% sucrose solution upon capture at the feeders. Bees were then kept at room temperature for 24 h inside an empty cooler with an open container of water to ensure humidity (~27°C, 60%–70% RH). After this starvation period, we randomly assigned bees to two groups: one that we fed again to satiation and one that we did not (hereafter, “unfed”). We transferred bees to the desiccation apparatus described above. Fed bees were, on average, 26.5% heavier and survived twice as long as unfed bees (Mass: 141.7 mg vs. 112 mg; Survival time: 14.2 h vs. 7.6 h, respectively). While the difference in survival time was significant (one‐way ANOVA, *F*_1,23_ = 7.37, *p* = 0.012), the water loss rate (*F*_1,21_ = 0.48, *p* = 0.496) and water content at mortality (*F*_1,23_ = 1.55, *p* = 0.226) were similar between feeding conditions (Figure S1). Many bees lack the ability to extend their proboscis and feed once food is presented directly, unlike honey bees and bumble bees, which can often be fed individually using this approach (Vorel and Pitts‐Singer 2010; Gonzalez et al. 2024). Thus, to standardize pre‐assay access to food across species, we provided all bees with *ad libitum* access to a drop of 50% sucrose solution placed at the bottom of the vial for 30 min before transferring them to the desiccation apparatus.

### 2.4 Morphological Traits

We assessed the influence of body size and hairiness on desiccation‐related traits. We used fresh body mass as a proxy of body size, and measured it before and after the desiccation tolerance assay to calculate the gravimetric water loss rate (WLR) as the percentage of mass assumed to be body water content lost over time. Critical water content (CWC) was estimated as the percentage of body water content at mortality, determined by subtracting the dry mass (Botsch et al. 2024). We measured dry mass after placing bees in a desiccation oven at 70°C for 3 days. As in Peters et al. (2016), we used the maximum hair length along the anterolateral corners of the mesoscutum as a proxy of body hairiness. We chose this region because it covers the flight muscles responsible for endogenous heat production, and hairs are typically short and sparse on the disc of the mesoscutum. We took all measurements with an ocular micrometer on an S6E stereomicroscope (Leica Microsystems, Wetzlar, Germany). Voucher specimens have been deposited in the Melissotheque of the Aegean.

### 2.5 Ecological Traits

To explore the influence of social behavior and nesting ecology on desiccation‐related traits, we assigned each species to the following categories: Social behavior was divided into strictly solitary, socially polymorphic, and eusocial. Socially polymorphic includes species with variable social behavior, such as those in the family Halictidae (i.e., species in the genera *Halictus* Latreille and *Lasioglossum* Curtis), which can range from solitary to social both within and among populations across their distribution range (e.g., Richards 2001; Michener 2007). Nesting guilds were divided into three categories: strictly ground nesting, preexisting cavities or constructed holes, and large comb‐building cavity nesters. Finally, we categorized species based on the nest location into three categories: above ground, below ground, or both. Bees using preexisting cavities or constructed holes, such as those in the family Megachilidae (e.g., *Osmia* Panzer), can nest either below or above ground (Müller 2018), so we separated them into the latter category. A single solitary species, *Anthidiellum breviusculum* (Pérez), builds aerial, exposed nests (Michener 2007). For analysis of the nesting guilds, we exclude aerial nests because of the limited number of species and specimens sampled. We extracted biological information from the literature (Paxton et al. 2002; Rust et al. 2004; Ulrich et al. 2009; Grace 2010; Gogala 2012; Praz 2017; Müller 2018; Mikát et al. 2019, 2022; Gonzalez et al. 2020; see Table S1) and, when unavailable, we inferred it from closely related species, as discussed by Michener (2007).

### 2.6 Statistical Analyses

We conducted all statistical analyses in R (R Core Team 2025). First, to evaluate how morphological and physiological traits covary, we performed a principal component analysis (PCA) on the following variables: fresh body mass, hair length, water loss rate (WLR), critical water content (CWC), and survival time following exposure to a desiccant. We used the prcomp function, excluded individuals with missing values, and standardized all variables prior to analysis (mean = 0, SD = 1) to ensure comparability across measurements with different scales. Then, to visualize individual scores along principal components according to nesting guild, nest location, and sociality, we used the fviz_pca_ind function in the factoextra package (Kassambara and Mundt 2020). We added 95% confidence ellipses to illustrate group dispersion and assessed the association between ecological categorical traits and PCA structure using the envfit function from the vegan package (Oksanen et al. 2022). This procedure tests whether ecological factors are significantly associated with variation along ordination axes using permutation tests. We performed 9999 permutations to obtain robust *p*‐values and set a random seed for reproducibility. To statistically test whether ecological groups differed significantly in the multivariate trait space, we performed a complementary permutational multivariate analysis of variance (PERMANOVA) using the adonis2 function from the vegan package.

To explore the drivers of desiccation tolerance, guided by patterns suggested by the PCA results (see below), we tested whether WLR and CWC predict survival time under desiccating conditions, both independently and in combination with fresh body mass. We first implemented linear mixed‐effect models (LMMs) to assess the effects of WLR and CWC individually and jointly, including collection date and species identity as random effects to account for the nonindependence among observations. We then extended the models to include fresh body mass and all interactions. Based on model comparison using the Akaike's Information Criterion (AIC), models including body mass and CWC substantially improved fit. Accordingly, we built a full model with all main effects and interactions and used the buildmer package (Voeten 2023) to simplify the model via backward selection, excluding nonsignificant interactions while maintaining AIC‐supported fit. The final simplified model is presented in the results.

To explore how morphological traits influence water balance physiology, we tested whether body mass and hair length predict WLR and CWC, both independently and in combination. For each response variable, we implemented LMMs initially including each morphological trait independently and accounting for species identity and collection date as random effects. We then expanded the models to include both morphological traits and their interactions. Model comparison using AIC was used to select the best‐fitting model for each response variable.

To assess whether survival time, WLR, and CWC differ among ecological categories (nesting guild, nest location, and sociality), we implemented LMMs for each of the three desiccation‐related response variables. Given that Fisher's exact tests revealed strong, significant associations among the selected ecological traits (*p* < 0.001 for all pairwise comparisons; see distribution of trait categories across the dataset in Figure S2), separate models were fitted for each predictor as a fixed effect, while controlling for body mass. We assessed the overall significance of predictors using a Type II Wald chi‐square test with the car package (Fox and Weisberg 2019), while effect sizes and directions were estimated from the model coefficients. In all models, we used collection date and species identity as random effects to account for the nonindependence among observations. To meet assumptions of normality, all variables were log‐transformed prior to analysis, except for CWC that was arcsine transformed and hair length, which follows a normal distribution and was analyzed on its original scale. Distributions and pairwise relationships among variables were visually inspected using the ggpairs function from the GGally package (Schloerke et al. 2024) (Figures S3 and S4).

Finally, because seven species were represented by a single individual, we repeated all LMMs after excluding these species to evaluate the robustness of the results obtained from the full dataset. In addition, we conducted phylogenetic generalized linear mixed models (PGLMMs) using the R package phyr (Li et al. 2020) to determine whether the factor effects are robust to phylogenetic associations.

### 2.7 Phylogenetic Signal

To assess the phylogenetic signal of desiccation‐related traits, we calculated Pagel's λ (Pagel 1999) for survival time, WLR, and CWC using the phylosig function from the phytools R package (Revell 2012). We used 10,000 simulations and a likelihood ratio test to assess significant departure from 0 (no phylogenetic signal). We used a pruned tree from the supermatrix phylogeny of bees (Henríquez‐Piskulich et al. 2024) that included most of our species except for *Ceratina parvula* Smith and *Osmia bidentata* Morawitz, which were replaced by their closest relatives ( *Ceratina braunsi* Eardley & Daly and *Osmia scutellaris* Morawitz). Three unidentified species in our study were excluded from the analysis (*Ceratina* sp., *Ceratina* sp. 2, and *Andrena* sp.). Thus, the final tree consisted of 19 taxa. We visualized trait values across phylogeny using the contMap function from the phytools.

## 3 Results

**FIGURE 1:**
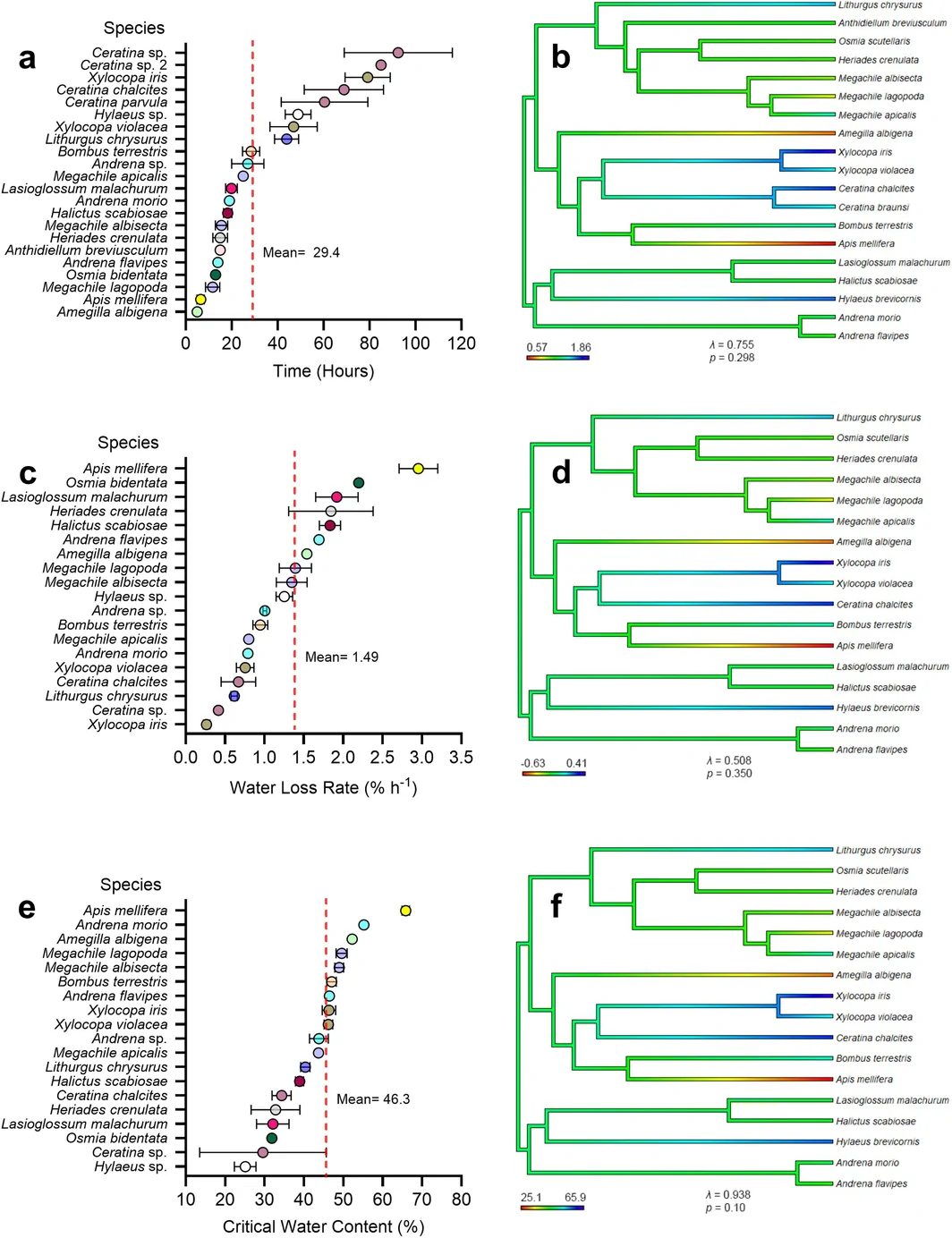
Distribution of data (left figures) and phylogenetic signal (right figures) of survival time under desiccating conditions (hours alive), water loss rate (% h^−1^), and critical water content (%) among 22 species of bees from the Greek island of Lesbos. (a, b) Survival time; (c, d) water loss rate; (e, f) critical water content. Red dotted line indicates the mean.

All desiccation‐related metrics and morphological traits varied greatly among bees in our study (Table 1). The survival time of bees exposed to a desiccant varied greatly among species (Figure 1a). On average, bees survived from as short as 5 h [ *Amegilla albigena* (Lepeletier)] to as long as 92.5 h (*Ceratina* sp.); WLR ranged from as low as 0.266% h^−1^ in *Xylocopa iris* (Christ) to as high as 2.96% h^−1^ in *Apis mellifera* Linnaeus (Figure 1); and CWC ranged from as low as 25% in *Hylaeus* sp. to as high as 65.9% in *A. mellifera* (Figure 1e). Fresh body mass ranged from an average of 3.4 mg in *Ceratina parvula* Smith to 632.8 mg in *Xylocopa violacea* (Linnaeus), while hair length ranged from an average of 0.06 mm in *Hylaeus* sp. to 1.83 mm in *Bombus terrestris* (Linnaeus).

**FIGURE 2:**
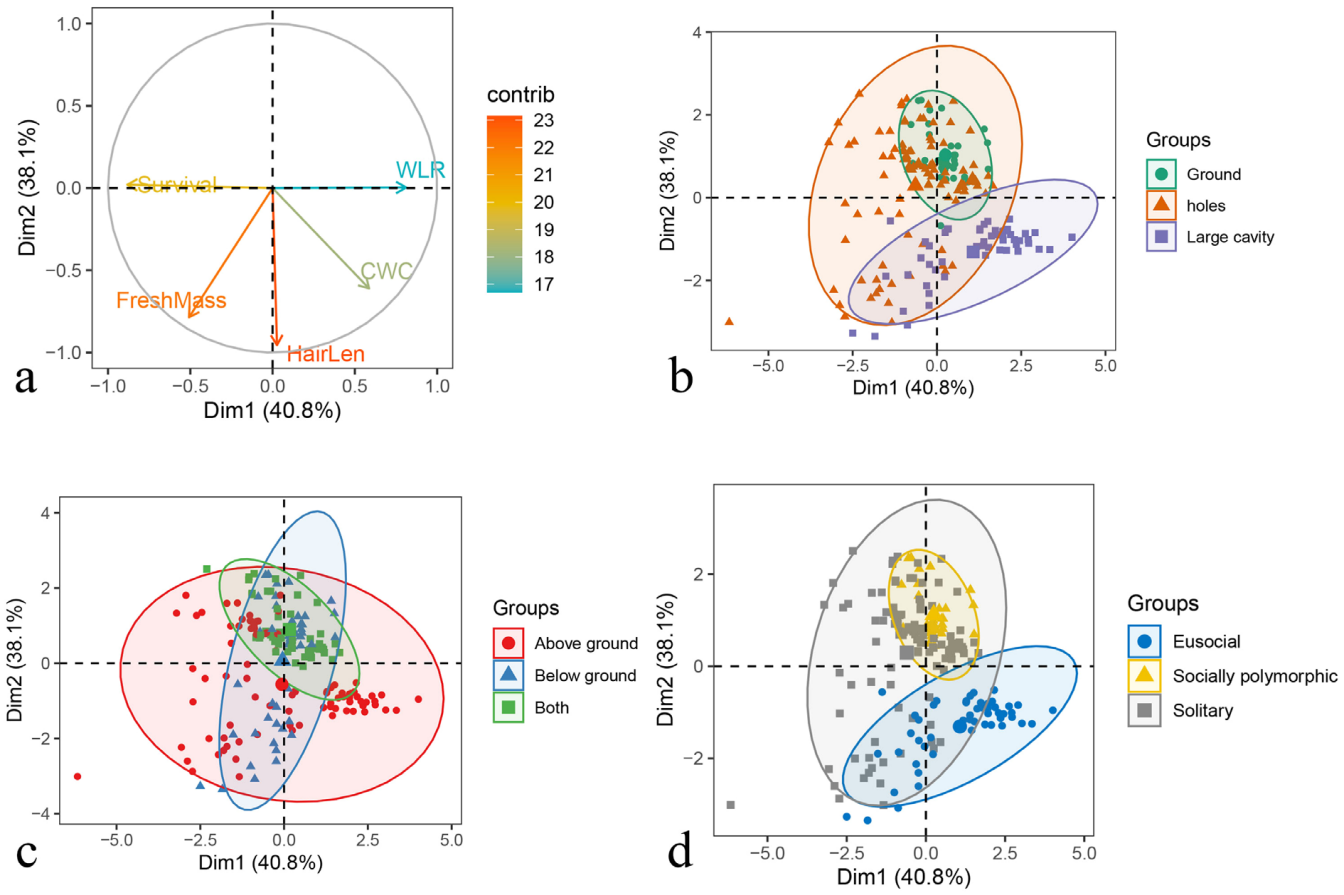
Principal component analysis (PCA) of desiccation‐related morphological and physiological traits. (a) PCA loading plot showing the contribution of traits to the first two principal components (PC1 = 40.8%, PC2 = 38.1%). Arrows represent the direction and magnitude of trait loadings for hair length (HairLen), body mass (FreshMass), survival time, water loss rate (WLR), and critical water content (CWC). Color intensity indicates the relative contribution of each variable to the principal components. (b–d) PCA scores grouped by nesting guild, nest location, and sociality, respectively with 95% confidence ellipses representing dispersion of groups in PCA space. Each point represents an individual bee and the number of individuals and species represented in each group are provided in Table S4. The PCA included 191 individuals representing 19 species after observations with missing data were excluded.

The principal component analysis revealed that the first two principal components explained 78.86% of the total variation among species (PC1: 40.81%; PC2: 38.05%). PC1 captured a water balance trade‐off, contrasting longer survival time (−0.618) with higher water loss rates (0.568). PC2 was primarily driven by morphology, with strong negative loadings for both hair length (−0.692) and body mass (−0.570) (Figure 2a, Table 2).

**TABLE 2:** Summary of the PCA loadings of variables on principal components (PC).

PCA variable	PC1	PC2	PC3	PC4	PC5
Hair length	0.019	−0.692	−0.162	0.059	−0.700
Fresh mass	−0.355	−0.570	−0.276	0.254	0.639
Survival time	−0.618	0.015	−0.111	−0.775	−0.071
WLR	0.568	0.003	−0.724	−0.362	0.150
CWC	0.411	−0.442	0.601	−0.448	0.272

*Note:* The first two principal components explained 78.86% of the total variation among species (PC1: 40.81%; PC2: 38.05%).

Abbreviations: CWC, Critical water content; WLR, Water loss rate.

Mapping ecological categories onto the PCA space revealed distinct clustering of functional traits (Figure 2b–d). Eusocial species, namely honey bees and bumble bees, that nest in large cavities occupied a distinct physiological space characterized by higher body mass, longer hair, and higher WLR. Conversely, solitary species, particularly those nesting in holes or below ground, were generally associated with longer survival times under desiccating conditions and smaller morphological profiles. Permutational tests (envfit) confirmed that the distribution of physiological and morphological traits in the PCA space was significantly structured by nesting guild (*R* ^2^ = 0.321, *p* = 0.001), nest location (*R* ^2^ = 0.093, *p* = 0.001), and sociality (*R* ^2^ = 0.320, *p* = 0.001). Complementary PERMANOVA analyses confirmed that nesting guild (*R* ^2^ = 0.268, *p* < 0.001) and nest location (*R* ^2^ = 0.221, *p* < 0.001) explained a significant portion of the multivariate trait differences among species, but sociality had a smaller, nonsignificant effect (*R* ^2^ = 0.005, *p* = 0.122). These results confirm that the distribution of traits in the PCA space is largely structured by nesting guild and location, mirroring patterns observed in the PCA and envfit analyses.

### 3.1 Effect of Water Balance Physiology and Body Mass on Survival Time

Survival time following exposure to a desiccant was strongly influenced by both WLR and CWC, as well as body mass. The best‐fitting model that described the observed variance in survival time included the main effects (WLR, body mass, CWC) and the interaction between CWC and body mass. Survival time decreased with increasing WLR (β = −0.97, *p* < 0.001) and higher CWC (β = −3.37, *p* < 0.001), whereas larger bees survived longer (β = 0.63, *p* < 0.001). The interaction between body mass and CWC was significant (β = −0.90, *p* < 0.001), indicating that larger bees benefit more from low CWC than WLR. Random effects of species identity and collection date accounted for a small portion of the variation in survival time (SD = 0.108 and 0.090, respectively), indicating that fixed effects capture the major drivers of bee survival time (Table 3). Excluding species represented by a single individual yielded identical results (Table S5). Additional PGLMM analyses to account for phylogenetic relatedness supported these relationships: survival time decreased with increasing WLR (β = −0.36) and higher CWC (β = −0.34) and increased with body mass (β = 0.10) (*p* < 0.001 in all cases). Similar results were obtained by excluding species represented by a single individual (β = −0.36, −0.33, and 0.11, respectively; *p* < 0.001 in all cases).

**TABLE 3:** Results of the linear mixed‐effects model predicting survival time under desiccating conditions in 22 bee species as a function of water loss rate (Log_10_WLR), critical water content (ArcCWC), and body wet mass (Log_10_Mass).

Predictor	Estimate (β)	Std. error	df	*t*‐value	*p*
Intercept	3.108	0.164	166.13	18.95	**< 0.001**
Log_10_WLR	−0.965	0.038	185.46	−25.28	**< 0.001**
ArcCWC	−3.375	0.301	180.93	−11.20	**< 0.001**
Log_10_Mass	0.627	0.113	149.06	5.55	**< 0.001**
ArcCWC × Log_10_Mass	−0.899	0.209	177.54	−4.29	**< 0.001**

Random effects		
Group	Variance	SD
Species	0.012	0.108
Collection date	0.008	0.090
Residual	0.012	0.109

*Note:* The final best‐fitting model included all fixed effects and a single interaction, as indicated below. Species and collection date were included as random effects. Significant values are indicated in bold (*p* < 0.05).

### 3.2 Effect of Morphology on Water Balance Physiology

Model comparisons using AIC indicated that body mass alone provided the best fit (AIC = 27.99) for predicting WLR, whereas models including hair length alone (AIC = 46.14), both traits together (AIC = 31.99), or their interaction (AIC = 28.36) did not improve model performance (Table S2). In the best model, WLR decreased significantly with increasing body mass (β = −0.447, *p* < 0.001). These results suggest that WLR is mainly determined by body mass rather than hairiness, with larger bees losing water more slowly than smaller bees. In contrast, CWC was not strongly associated with body mass. Model comparisons indicated that the model including hair length alone provided the best fit (AIC = −371.20) compared to the other candidate models (Table S2). In this model, CWC increased significantly with increasing hair length (β = 0.101, *p* < 0.001). These results suggest that hair length is the primary morphological trait associated with variation in CWC among species, with hairier bees exhibiting higher CWC values in our sample. Excluding species represented by a single individual did not affect model selection or the main morphological relationships (Table S6). These relationships were also supported after accounting for phylogenetic relatedness in the PGLMMs: WLR decreased with increasing body mass (β = −0.25) whereas CWC increased with increasing hair length (β = 0.07; *p* < 0.001 in both cases). Similar results were obtained after excluding species with a single individual (Body mass: β = −0.26, *p* < 0.001; Hair length: β = −0.26, *p* = 0.002).

### 3.3 Nesting and Social Traits

**FIGURE 3:**
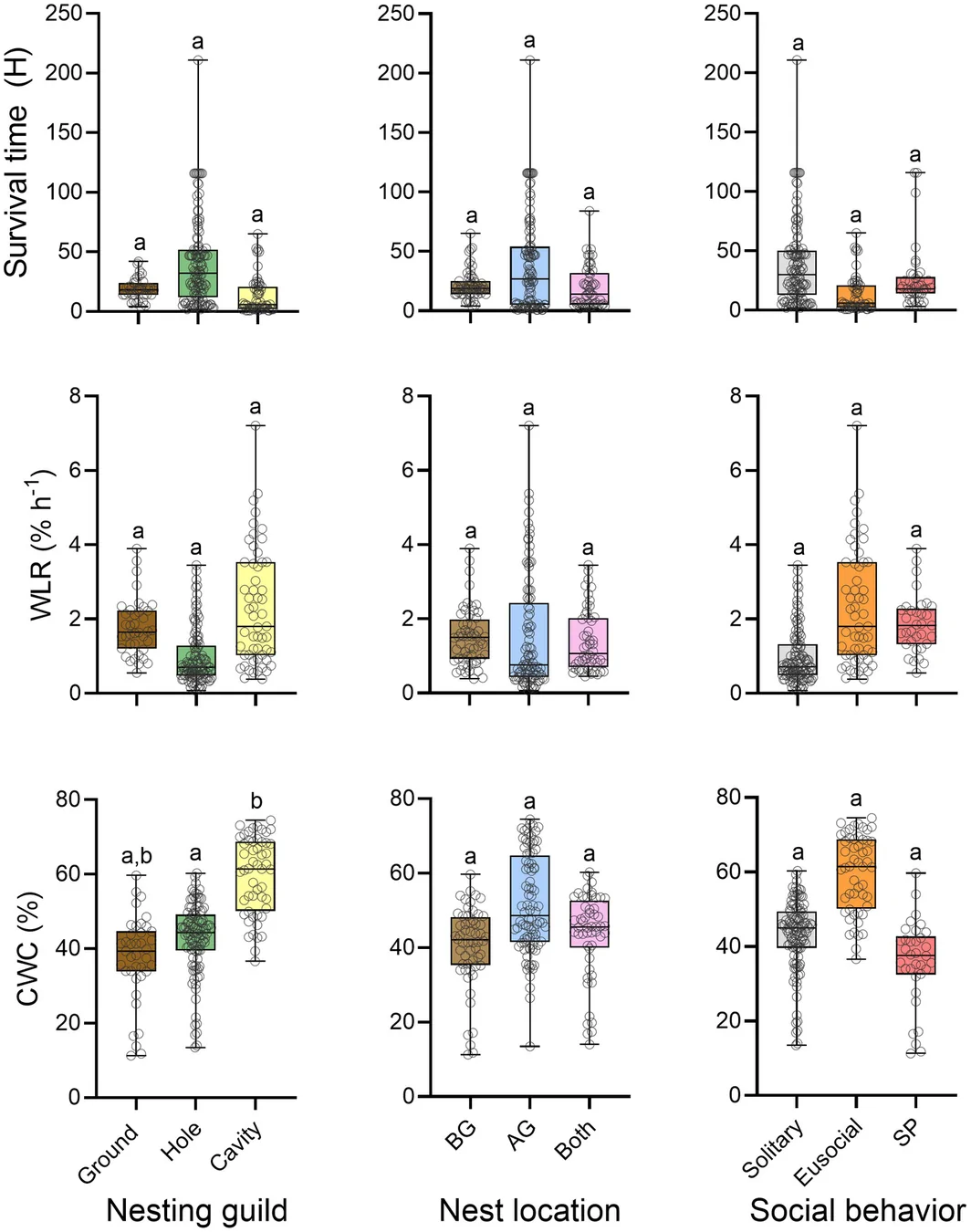
Survival time, water loss rate (WLR), and critical water content (CWC) discriminated by nesting guild, nest location, and social behavior. Nesting guild: Ground = strictly ground nesters; Hole = nesting in preexisting cavities or constructed holes; Cavity = large comb‐building cavity nesters. Nest location: BG = below ground; AG = above ground; Both = nesting above and below ground. Social behavior: Solitary = strictly solitary species; Eusocial = eusocial species; SP = socially polymorphic species. See text for further explanation. Box plots show medians, quartiles, and extreme values. For each figure, a different letter above bars indicates significant differences (*p* < 0.05).

Bee survival time, WLR, and CWC varied among nesting and sociality traits (Figure 3, Table 4, Table S3). After controlling for body mass, LMMs showed that nesting guild significantly influenced WLR (*χ* ^2^ = 8.19, *p* = 0.017) and CWC (*χ* ^2^ = 7.71, *p* = 0.021), while its effect on survival time was marginal (*χ* ^2^ = 5.92, *p* = 0.052). However, post hoc Tukey comparisons detected no significant differences in WLR or survival time among nesting guilds (all *p* > 0.05). In contrast, pairwise comparisons revealed that hole‐nesting species had significantly lower CWC than large cavity‐nesting bees (β = −0.192 ± 0.071 SE, *p* = 0.049), whereas ground‐nesting species did not differ significantly from the other groups (Figure 3, Table 4). While sociality did not significantly influence survival time or WLR, it was a significant predictor of CWC (*χ* ^2^ = 7.36, *p* = 0.025). However, pairwise comparisons revealed only marginal differences between eusocial and socially polymorphic species (*p* = 0.071) and between eusocial and solitary species (*p* = 0.078), while socially polymorphic and solitary species did not differ (*p* = 0.646). Nest location did not significantly affect any of the desiccation‐related traits. These results suggest that nesting guild influences water balance physiology (WLR and CWC), whereas social organization affects dehydration thresholds but not WLR or survival time. Excluding species represented by a single individual also yielded broadly consistent results, although sociality was significantly associated with survival time, WLR, and CWC (Table 4, Table S7). After accounting for phylogenetic relatedness, PGLMMs supported the effects of nesting guild on WLR (*χ* ^2^ = 7.87, *p* = 0.020) and sociality on survival time (*χ* ^2^ = 6.14, *p* = 0.05); remaining ecological factors were nonsignificant (*p* > 0.05). However, only the effect of sociality on survival time remained after excluding species represented by a single individual (Sociality: *χ* ^2^ = 6.85, *p* = 0.03; Nesting guild: *χ* ^2^ = 5.78, *p* = 0.06).

**TABLE 4:** Type II Wald Chi‐square tests for fixed effects in linear mixed‐effects models assessing the influence of ecological traits (nesting guild, nest location, and sociality) on survival time, water loss rate (WLR), and critical water content (CWC).

Response	Factor	Full dataset		Reduced dataset	
		Chi‐square	*p*	Chi‐square	*p*
Survival time	Nesting guild	5.917	0.052	4.523	0.104
	Nest location	3.140	0.208	0.219	0.896
	Sociality	5.068	0.079	7.907	**0.019**
WLR	Nesting guild	8.191	**0.017**	6.446	**0.040**
	Nest location	2.583	0.275	0.938	0.626
	Sociality	5.875	0.053	6.813	**0.033**
CWC	Nesting guild	7.706	**0.021**	7.683	**0.022**
	Nest location	1.317	0.518	0.787	0.675
	Sociality	7.360	**0.025**	8.654	**0.013**

*Note:* Results are presented for analyses including 22 species (full dataset) and excluding 7 species represented by a single specimen (reduced dataset). Models included fresh mass as a covariate and species identity and collection date as random effects. Significant values are indicated in bold (*p* < 0.05). Degrees of freedom were two in all cases.

### 3.4 Phylogenetic Signal

Pagel's values suggest varying degrees of phylogenetic signals across traits, although none were statistically significant (Figure 1b,d,f). These values ranged from moderate in WLR (*λ =* 0.508, *p* = 0.350), strong for survival time (*λ* = 0.755, *p* = 0.298), to very strong in CWC (*λ =* 0.938, *p* = 0.10).

## 4 Discussion

Our study is the first to describe how some desiccation tolerance metrics vary among a morphologically and ecologically diverse assemblage of Mediterranean bees. Using multivariate analyses with mechanistic models, we show that survival time under acute desiccating conditions, WLR, and CWC vary significantly among species, and that these are shaped by a combination of morphology, ecology, and physiological traits. Understanding these traits is critical for predicting species vulnerability under increasingly arid conditions driven by climate change.

The PCA analysis (Figure 2a) revealed that desiccation‐related traits are structured along two major axes corresponding to physiological water balance (PC1, survival time, and WLR) and morphological variation (PC2, body mass and hair length). Consistent with these patterns observed in the PCA, our models revealed that survival time was strongly influenced by body mass, WLR, and CWC. As expected, larger bees survived longer under desiccating conditions, likely due to lower surface area‐to‐volume ratios, increased water storage, and higher lipid reserves that could be converted to metabolic water (e.g., Edney 1977; Hadley 1994; Chown et al. 1998; Nervo et al. 2021). Survival time decreased with increasing WLR and higher CWC, indicating that both the rate at which water is lost and the dehydration threshold influence survival time. In addition, the significant interaction between body mass and CWC suggests that body size modifies how dehydration thresholds affect survival time, highlighting the likely multifaceted nature of desiccation tolerance in bees.

Our models showed how morphological traits influenced these physiological mechanisms. WLR decreased significantly with increasing body mass, suggesting that body size is an important determinant of evaporative water loss in bees. In contrast, hair length was strongly associated with CWC, suggesting that hairiness may influence dehydration thresholds rather than the rate at which water is lost (Table S2). The latter result is unexpected given that hairiness is thought to help reduce evaporative water loss by trapping a layer of humid air or protecting against convective airflow (Buxton et al. 2021). However, our study assessed only hair length, a commonly used trait in bee studies due to its ease of measurement (Ostwald, Gonzalez, et al. 2024) and its association with cooler climates (Peters et al. 2016; Gonzalez et al. 2022). Nonetheless, hairiness is a multidimensional trait that includes other features that may also influence desiccation. These comprise hair structure, density, orientation, thickness, color, and spatial distribution of hairiness, which can vary even within individuals (e.g., Pasteels and Pasteels 1971; Michener 2007; Hines et al. 2022). For example, both hair density and color in bees have been linked to variation in both ambient temperature and precipitation (Suni and Dela Cruz 2021; Ostwald et al. 2025). Future studies should incorporate these dimensions to better understand the relationship between hairiness and desiccation tolerance in bees.

Other morphological traits may also be important but remain understudied in bees. Cuticular permeability, determined by hydrocarbon composition, thickness, and surface structure, is a major determinant of water loss rates in insects (Gibbs 1998). Body shape, spiracle size, and wing area may also be relevant (e.g., Chown et al. 1998; Arcaz et al. 2016; Castro et al. 2021; Nervo et al. 2021). Bees vary considerably in body shape and spiracle morphology (Hatjina et al. 2004; Michener 2007), and these features may affect surface area or respiratory water loss, but their functional significance remains largely unexplored.

Mapping ecological traits onto the multivariate space showed that nesting guild, nest location, and sociality contributed to structuring this trait variation (Figure 2b–d). Eusocial species nesting in large cavities tended to occupy a distinct region of trait space characterized by larger body size, longer hair, and relatively higher WLR, whereas solitary species, especially those nesting in holes or below ground, tended to cluster toward longer survival time and smaller body size. Complementary PERMANOVA analyses supported these patterns, with nesting guild and nest location explaining significant portions of the multivariate trait variation. Consistent with these patterns suggesting that ecological strategies shape combinations of physiological and morphological traits, our models revealed that ecological traits influence the physiological mechanisms underlying desiccation tolerance.

Although nesting guild, nest location, and sociality did not significantly affect survival time directly, nesting guild influenced WLR and CWC, and sociality influenced CWC (Figure 3, Table 4, Table S3). Hole‐nesting species exhibited lower CWC than species nesting in large cavities, suggesting that nesting strategy may shape dehydration thresholds. Eusocial species tended to have higher CWC values than solitary species, although pairwise differences were only marginally significant (Figure 3). These trends are consistent with the more stable, humid, and buffered environments of below ground nests or with the regulated microclimates created by collective nesting in eusocial species. Similar results have been reported in other insects, where habitat or microhabitat strongly influences desiccation traits, with species or populations from drier environments typically exhibiting lower metabolic rates and reduced water loss (e.g., Harrison et al. 2012; Bujan et al. 2016; Kellermann and van Heerwaarden 2019).

In eusocial bees, higher CWC may be facilitated by larger body size, regulated nest environments, and continuous access to food and water through stored resources and trophallaxis. For instance, honey bees and bumble bees, maintain relatively constant nest humidity by fanning or transporting water (e.g., Weidenmüller et al. 2002; Jones and Oldroyd 2006; Ellis et al. 2008). Trophallaxis, the mouth‐to‐mouth transfer of food, symbionts, and nutrients between adults and immatures, or among adults (Wcislo and Gonzalez 2006; Michener 2007), is another feature of eusocial bees that may help sustain water balance. Together, these features likely contribute to higher body water content and influence how eusocial bees cope with desiccation stress. Our results also suggest that the solitary, ground‐nesting species examined here may face greater desiccation risk under climate change, which is reducing water availability and accelerating soil drying (Berg and Sheffield 2018).

Although only a few studies have examined desiccation tolerance and related physiological traits in bees, some patterns are emerging. First, bees vary considerably in WLR, CWC, and survival time under desiccating conditions, potentially helping identify susceptible species. For example, the relatively high WLR and CWC values of *A. mellifera* and *B. terrestris* in Greece suggest susceptibility to dehydration. These estimates are similarly high to those reported for *A. mellifera* and *B. impatiens* in North America, although absolute values are different (Burdine and McCluney 2019; Botsch et al. 2024). Second, life‐history traits appear to contribute to this variation. The WLR and CWC of twig‐nesting bees *Ceratina*, *Xylocopa*, and *Hylaeus* (Figure 1c,e) may help explain their long survival time in our assays and their ability to exploit sun‐exposed habitats or nesting substrates that provide little insulation from environmental extremes (Gonzalez et al. 2020, 2023). This explains the greater desiccation resistance of invasive twig‐nesting bees relative to an endemic ground‐nesting bee in Fiji (da Silva et al. 2021). Third, behavioral and social mechanisms may buffer individual physiological susceptibility. Honey bees mitigate thermal and desiccation stress by adjusting foraging schedules, fanning the nest, and transporting water (Jones and Oldroyd 2006; Ellis et al. 2008; Gonzalez et al. 2024). Solitary bees may also adjust their activity periods to prevent excessive water loss (Johnson, Alvarez, and Harrison 2023), while social contact can reduce water loss even in solitary species (Ostwald, Venegas, and Seltmann 2024). Together, our results highlight significant differences between solitary and eusocial bees in desiccation tolerance and its underlying mechanisms, providing insights into how bee distributions and community composition may shift under ongoing environmental change.

Our phylogenetic analyses revealed moderate to strong phylogenetic signals for all desiccation‐related traits, especially for CWC, although none of these signals were statistically significant. Thus, these results partially support our hypothesis that closely related species tend to exhibit similar physiological responses to desiccation stress. The lack of statistical significance in our study is likely due to the small number of specimens and species used (Table 1), as well as the small geographical scale of our study, as discussed for studies with ants assessing thermal traits (Nascimento et al. 2022). In addition, our study was restricted to an assemblage of summer species. Future studies should address this not only by including more species and representatives from other lineages but expanding to other bee faunas from other regions. Assessing species from the humid tropics would provide a broader and more comprehensive evaluation of desiccation tolerance in bees, given that these species are predicted to exhibit lower desiccation resistance and these regions are dominated by stingless bees, a major species‐rich clade of eusocial species.

It is worth mentioning that the relative importance of these physiological, morphological, and ecological drivers may depend on the scale of analysis (e.g., Pickert et al. 2026). For example, at a local scale, activity period or nest‐site selection can modify climatic exposure (e.g., da Silva et al. 2026) and may buffer bees with relatively high WLR or CWC from desiccation stress. However, across broader climatic gradients, physiological or morphological traits may become more relevant in shaping species' climatic niches and distributions. Thus, the patterns observed in our experiments require validation across environmental, geographic, or phylogenetic scales.

Our experimental approach also has several limitations that should be considered when interpreting these results. First, we used a desiccation apparatus employed in previous studies of ants and bees (Bujan et al. 2016; Gonzalez et al. 2023, 2024), which may have introduced confounding factors such as hypoxia, starvation, and social isolation. Our setup is a closed system that prevents airflow and does not allow continued feeding during the assay. Bees were also tested individually, regardless of social origin, despite evidence that social contact can reduce WLR in bees under experimental conditions (Ostwald, Venegas, and Seltmann 2024). Second, because we aimed to maximize the taxonomic representation of field‐collected specimens, we did not include a non‐desiccant control. The absence of control treatments prevented us from determining how much mortality resulted from dehydration rather than from limitations of the experimental procedure. However, studies of ants have demonstrated that individuals in control treatments lived significantly longer than those exposed to desiccating conditions (Bujan et al. 2016). Furthermore, our measurements of WLR and CWC also confirm that individuals lost body mass during the assays. Similar experiments with bees have detected effects of desiccating conditions on survival time in some species but not in others (e.g., Gonzalez et al. 2023; Botsch et al. 2024). Thus, without control treatments, we cannot attribute the observed survival times entirely to desiccation. These experimental factors may have shortened survival times, but the apparatus is simple and reliable in the field, facilitating the assessment and comparison of multiple taxa across regions.

Our pilot experiment with honey bees indicated that survival time is highly dependent on the feeding status of individuals, which is difficult to standardize in field‐collected specimens. These results suggest that while survival time is a practical metric, especially for field studies, other desiccation‐related metrics, namely WLR and CWC, may provide more reliable and consistent indicators of physiological tolerance. However, measuring body mass for very small bees (some species of *Hylaeus*, *Ceratina*, *Perdita*, etc.) can be challenging with standard field equipment, making survival time a more accessible approach under such conditions. Finally, our assays under constant temperature likely do not reflect the interacting and fluctuating thermal and hydric conditions encountered by bees in nature. Given that temperature influences vapor‐pressure deficit, metabolic demand, respiratory water loss, and potentially cuticular water loss (Botsch et al. 2024; Sinclair et al. 2024), species' responses may change under elevated or fluctuating temperatures. Future studies should validate these responses across realistic temperature and humidity regimes to obtain more accurate predictions of vulnerability to desiccation under field conditions.

In conclusion, our results indicate that desiccation tolerance in bees is shaped by a complex interaction of physiological, morphological, and ecological factors, and likely influenced by evolutionary history. Body size strongly influences both WLR and survival time, whereas hairiness appears to affect CWC rather than water loss directly. Ecological traits such as nesting guild and social organization further influence physiological mechanisms underlying desiccation tolerance, even when their direct effects on survival time are limited. These findings suggest that small, ground‐nesting solitary bee species with high WLR may be vulnerable to changes in precipitation and increased aridity under climate change. Understanding these vulnerabilities is essential for predicting how bee communities may respond to shifting environmental conditions.

Given that our study was based on a single regional assemblage sampled on one island over a narrow temporal window, and desiccation tolerance can vary spatially and temporally (Li and Heinz 1998; Sjursen and Sømme 2000; Kellermann and van Heerwaarden 2019), these patterns require validation across broader spatial, temporal, and phylogenetic scales. In addition, seven species were each represented by a single individual (Table 1). Excluding these species from the analyses yielded broadly consistent results with those from the full dataset, although ecological factors were more sensitive to their exclusion than physiological and morphological relationships (Table 4 and Tables S5–S7). PGLMMs also supported the main physiological and morphological relationships after accounting for phylogenetic relatedness but provided support only to the effects of nesting guild on WLR and sociality on survival time among the ecological traits. The small number of species within each ecological category (Table S4) may have contributed to these results. Nevertheless, further studies should address these limitations by increasing taxonomic representation and by sampling across greater spatial and temporal scales.

## Author Contributions

**Victor H. Gonzalez:** conceptualization (lead), data curation (lead), funding acquisition (equal), investigation (equal), methodology (equal), project administration (equal), writing – original draft (lead), writing – review and editing (lead). **Anna Dreusicke:** investigation (equal), methodology (equal), writing – review and editing (equal). **Meredith G. Johnson:** conceptualization (equal), investigation (equal), methodology (equal), writing – review and editing (equal). **Thomas Tscheulin:** funding acquisition (equal), investigation (equal), writing – review and editing (equal). **Theodora Petanidou:** funding acquisition (equal), investigation (equal), writing – review and editing (equal). **John M. Hranitz:** funding acquisition (equal), investigation (equal), methodology (equal), writing – review and editing (equal). **John F. Barthell:** funding acquisition (equal), investigation (equal), methodology (equal), project administration (equal), writing – review and editing (equal).

## Funding

This work was supported by a grant from the National Science Foundation's REU program (EAR 1950805) to the University of Central Oklahoma (to C.K. Simmons). Open Access publishing of this article was supported by the David Henry Wenrich Memorial Endowment fund at the University of Kansas.

## Conflicts of Interest

The authors declare no conflicts of interest.

## Supporting information


**Figure S1:** Feeding status and desiccation tolerance in honey bees. This pilot study assessed the effect of food access on survival time under simulated acute desiccation stress, water loss rate (WLR, % h^−1^), and critical water content (CWC, %). Comparison of fed (*n* = 13) and unfed (*n* = 12) bees in (a) initial body mass, (b) survival time, (c) water loss rate, and (d) critical water content. Box plots show median, quartiles, and extreme values. For each figure, a different letter above bars indicates significant differences (*p <* 0.05).
**Figure S2:** Alluvial plot showing the associations among nesting guilds, sociality, and nest location. The width of each stream represents the frequency of species sharing the same combination of ecological traits. Strata represent individual trait categories, while alluvia illustrate the flow of trait combinations across axes. Colors indicate nesting guilds. Nesting guild: Ground = strictly ground nesters; Hole = nesting in preexisting cavities or constructed holes; Cavity = large comb‐building cavity nesters. Nest location: below ground, above ground or both. Social behavior: solitary, eusocial, and socially polymorphic species.
**Figure S3:** Pairwise plot matrix showing the distribution and relationships among model variables. Density plots are displayed along the diagonal, scatterplots in the lower left, and correlation coefficients in the upper right. Variables: Survival = bee survival time (hours); WLR = water loss rate (% h^−1^); CWC = critical water content (%); FreshMass = wet mass (g); DryMass = dry body mass (g); HairLen = hair length (mm).
**Figure S4:** Pairwise plot matrix showing the distribution and relationships among log_10_‐transformed and untransformed variables. Density plots are displayed along the diagonal, scatterplots in the lower left, and correlation coefficients in the upper right. Variables: L_10_Survival = Log_10_‐transformed bee survival time (hours); L_10_WLR = Log_10_‐transformed water loss rate (% h^−1^); ArcCWC = arcsine transformed critical water content (%); L_10_FreshMass = Log_10_‐transformed wet mass (g); L_10_DryMass = Log_10_‐transformed dry body mass (g); HairLen = hair length (mm).
**Table S1:** Life‐history, morphological, and desiccation‐related traits of bee species used in desiccation tolerance assays from the Greek island of Lesbos. Nesting guild (NG): A = Aerial; G = ground‐nesters; H = hole‐nesters; L = Large cavity nesters. Nest location (NL): AG = above ground; BG = Below ground; B = Both. Sociality (SL): E = Eusocial; P = Socially polymorphic; S = Solitary.
**Table S2:** Comparison of linear mixed models assessing the influence of body mass and hair length as predictors of water loss rate and critical water content across 22 bee species. For each response variable, we fitted models initially including each morphological trait independently, then expanding them to include both morphological traits and their interactions. Model comparison using Akaike's Information Criterion (AIC) was used to select the best‐fitting model for each response variable. Best models and significant effects are indicated in bold (*p* < 0.05). Species and collection date were used as random effects.
**Table S3:** Results of the linear mixed‐effects model assessing differences in survival time under desiccating conditions, water loss rate (WLR), and critical water content (CWC) among nesting guild, nest location, and sociality. This table presents the estimated coefficients, standard errors, *t*‐values, and *p*‐values for the fixed effects in models analyzing the response variables: Survival time, water loss rate (WLR), and critical water content (CWC). Factors such as nesting, nest location, and sociality were included in the models, with results for each factor provided.
**Table S4:** Number of individual bees and species represented in each nesting guild, nest location, and sociality group included in the principal component analysis. See also Figure 2.
**Table S5:** Results of the linear mixed‐effects model predicting survival time under desiccating conditions in 15 bee species as a function of water loss rate (Log_10_WLR), critical water content (ArcCWC), and body wet mass (Log_10_Mass). In these analyses, seven species represented by a single specimen were excluded. The final best‐fitting model included all fixed effects and a single interaction, as indicated below. Species and collection date were included as random effects.
**Table S6:** Comparison of linear mixed models assessing the influence of body mass and hair length as predictors of water loss rate and critical water content across 15 bee species. In these analyses, seven species represented by a single specimen were excluded. For each response variable, we fitted models initially including each morphological trait independently, then expanding them to include both morphological traits and their interactions. Model comparison using Akaike's Information Criterion (AIC) was used to select the best‐fitting model for each response variable. Best models and significant effects are indicated in bold (*p* < 0.05). Species and collection date were used as random effects.
**Table S7:** Results of the linear mixed‐effects model assessing differences in survival time under desiccating conditions, water loss rate (WLR), and critical water content (CWC) among nesting guild, nest location, and sociality across 15 species. In these analyses, seven species represented by a single specimen were excluded. This table presents the estimated coefficients, standard errors, *t*‐values, and *p*‐values for the fixed effects in models analyzing the response variables: Survival time, water loss rate (WLR), and critical water content (CWC). Factors such as nesting, nest location, and sociality were included in the models, with results for each factor provided.

## Data Availability

All relevant data and resources can be found within the article and its Supporting Information. The complete datasets used for the analyses in this study are available on Dryad: https://doi.org/10.5061/dryad.t1g1jwth9.
