# Supplementary material for: Morphological and Ecological Traits Explain Variation in Desiccation Tolerance Among Bees
Source: Ecol Evol. 2026 Sep 20;16(9):e74355. doi: 10.1002/ece3.74355 (PMC13590883; doi:10.1002/ece3.74355)
Supplement: Supplementary file 1 [file ECE3-16-e74355-s001.docx]

*Supplemental Materials*

**Morphological and ecological traits explain variation in desiccation tolerance among bees**

**Victor H. Gonzalez**^1*^**, Anna Dreusicke**^2^**, Meredith G. Johnson^3^, Thomas Tscheulin**^4^**, Theodora Petanidou**^4^**, John M. Hranitz**^5^**,** **and John F. Barthell**^6^

^1^ Biodiversity Institute and Department of Ecology and Evolutionary Biology, University of Kansas, Lawrence, Kansas, 66045, U.S.A.

https://orcid.org/0000-0002-4146-1634

^2^ Department of Biology, University of Iowa, 129 E Jefferson St, Iowa City, Iowa, 52245, U.S.A.

^3^ Department of Biological Sciences, North Dakota State University, Fargo, ND, 58281, U.S.A.

^4^ Laboratory of Biogeography and Ecology, Department of Geography, University of the Aegean, University Hill, Mytilene, 81100, Greece.

^5^ Department of Biology, Commonwealth University of Pennsylvania, Bloomsburg, Pennsylvania, 17815, U.S.A.

^6^ Department of Biology, University of Central Oklahoma, Edmond, Oklahoma, 73034, U.S.A.

*Author for correspondence ([vhgonza@ku.edu](mailto:vhgonza@ku.edu))

Running title: Desiccation tolerance among bees

**Figure S1.** Feeding status and desiccation tolerance in honey bees. This pilot study assessed the effect of food access on survival time under simulated acute desiccation stress, water loss rate (WLR, % h^-1^), and critical water content (CWC, %). (a) Comparison of fed (*n*=13) and unfed (*n*=12) bees in (a) initial body mass, (b) survival time, (c) water loss rate, and (d) critical water content. Box plots show median, quartiles, and extreme values. For each figure, a different letter above bars indicates significant differences (*p* *<*0.05).

**Figure S2.** Alluvial plot showing the associations among nesting guilds, sociality, and nest location. The width of each stream represents the frequency of species sharing the same combination of ecological traits. Strata represent individual trait categories, while alluvia illustrate the flow of trait combinations across axes. Colors indicate nesting guilds. Nesting guild: Ground= strictly ground nesters; Hole= nesting in pre-existing cavities or constructed holes; Cavity = large comb-building cavity nesters. Nest location: below ground, above ground or both. Social behavior: solitary, eusocial, and socially polymorphic species.

**Figure S3.** Pairwise plot matrix showing the distribution and relationships among model variables. Density plots are displayed along the diagonal, scatterplots in the lower left, and correlation coefficients in the upper right. Variables: Survival = bee survival time (hours); WLR = water loss rate (% h^-1^); CWC = critical water content (%); FreshMass = wet mass (g); DryMass= dry body mass (g); HairLen= hair length (mm).

**Figure S4.** Pairwise plot matrix showing the distribution and relationships among log_10_-transformed and untransformed variables. Density plots are displayed along the diagonal, scatterplots in the lower left, and correlation coefficients in the upper right. Variables: L10Survival = Log_10_-transformed bee survival time (hours); L10WLR = Log_10_-transformed water loss rate (% h^-1^); ArcCWC = arcsine transformed critical water content (%); L10FreshMass = Log_10_-transformed wet mass (g); L10DryMass= Log_10_-transformed dry body mass (g); HairLen= hair length (mm).

**Table S1.** Life-history, morphological, and desiccation-related traits of bee species used in desiccation tolerance assays from the Greek island of Lesbos. Nesting guild (NG): A = Aerial; G = ground-nesters; H = hole-nesters; L= Large cavity nesters. Nest location (NL): AG = above ground; BG = Below ground; B = Both. Sociality (SL): E = Eusocial; P = Socially polymorphic; S = Solitary.

| **Species** | **Life history** | | | **References** |
| --- | --- | --- | --- | --- |
|  | **NG** | **NL** | **SL** |  |
| Andrenidae |  |  |  |  |
| *Andrena flavipes* | G | BG | S | https://www.wildbienen.de/eb-aflav.htm |
| *Andrena morio* | G | BG | S | Gogala (2012) |
| *Andrena* sp. | G | BG | S | Michener (2007) |
| Apidae |  |  |  |  |
| *Amegilla albigena* | G | BG | S | Michener (2007); Grace (2010) |
| *Apis mellifera* | L | AG | E | Michener (2007) |
| *Bombus terrestris* | L | BG | E | Michener (2007) |
| *Ceratina chalcites* | H | AG | S | Mikát et al. (2019) |
| *Ceratina parvula* | H | AG | P | Mikát et al. (2022) |
| *Ceratina* sp. | H | AG | S | Michener (2007) |
| *Ceratina* sp. 2 | H | AG | S | Michener (2007) |
| *Xylocopa iris* | H | AG | S | Gonzalez et al. (2020) |
| *Xylocopa violacea* | H | AG | S | Gonzalez et al. (2020) |
| Colletidae |  |  |  |  |
| *Hylaeus* sp. | H | B | S | Michener (2007) |
| Halictidae |  |  |  |  |
| *Halictus scabiosae* | G | BG | P | Ulrich et al. (2009) |
| *Lasioglossum malachurum* | G | BG | P | Paxton et al. (2002) |
| Megachilidae |  |  |  |  |
| *Anthidiellum breviusculum* | A | AG | S | Michener (2007) |
| *Heriades crenulata* | H | B | S | Michener (2007) |
| *Lithurgus chrysurus* | H | AG | S | Rust et al. (2004) |
| *Megachile albisecta* | H | B | S | Praz (2017) |
| *Megachile apicalis* | H | B | S | Praz (2017) |
| *Megachile lagopoda* | H | B | S | Praz (2017) |
| *Osmia bidentata* | H | B | S | Müller (2018) |

**Table S2.** Comparison of linear mixed models assessing the influence of body mass and hair length as predictors of water loss rate and critical water content across 22 bee species. For each response variable, we fitted models initially including each morphological trait independently, then expanding them to include both morphological traits and their interactions. Model comparison using Akaike’s Information Criterion (AIC) was used to select the best-fitting model for each response variable. Best models and significant effects are indicated in bold (p < 0.05). Species and collection date were used as random effects.

| **Model** | **Fixed effects** | **DF** | **AIC** | **Estimate ± SE** | **T-value** | **p-value** |
| --- | --- | --- | --- | --- | --- | --- |
| Water Loss Rate | | | | | | |
| A | Mass | 5 | **27.99** | -0.447 ± 0.10 | -4.540 | **<0.001** |
| B | Hair length | 5 | 46.14 | -0.132 ± 0.09 | -1.452 | 0.153 |
| C | Mass + Hair length | 6 | 31.99 | -0.512 ± 0.12 (Mass) | -4.411 | **<0.001** |
|  |  |  |  | 0.120 ± 0.10 (Hair length) | 1.161 | 0.248 |
| D | Mass * Hair length | 7 | 28.36 | -0.208 ± 0.16 (Mass) | -1.325 | 0.190 |
|  |  |  |  | -0.176 ± 0.15 (Hair length) | -1.208 | 0.229 |
|  |  |  |  | -0.398 ± 0.14 (Interaction) | -2.912 | **0.005** |
| Critical Water Content | | | | | | |
| E | Mass | 5 | -366.5 | 0.029 ± 0.04 | 0.748 | 0.458 |
| F | Hair length | 5 | **-371.2** | 0.101± 0.03 | 3.082 | **<0.001** |
| G | Mass + Hair length | 6 | -365.0 | -0.020 ± 0.04 (Mass) | -0.469 | 0.640 |
|  |  |  |  | 0.110 ± 0.04 (Hair length) | 2.842 | **<0.001** |
| H | Mass * Hair length | 7 | -359.5 | 0.022 ± 0.06 (Mass) | 0.382 | 0.704 |
|  |  |  |  | 0.073 ± 0.06 (Hair length) | 1.33 | 0.187 |
|  |  |  |  | -0.050 ± 0.05 (Interaction) | -0.994 | 0.323 |

**Table S3**. Results of the linear mixed-effects model assessing differences in survival time under desiccating conditions, water loss rate (WLR), and critical water content (CWC) among nesting guild, nest location, and sociality. This table presents the estimated coefficients, standard errors, t-values, and p-values for the fixed effects in models analyzing the response variables: Survival time, Water loss rate (WLR), and critical water content (CWC). Factors such as nesting, nest location, and sociality were included in the models, with results for each factor provided.

| **Response** | **Predictor** | **Estimate (**β**)** | **S.E.** | **df** | **t-value** | **p-value** |
| --- | --- | --- | --- | --- | --- | --- |
| Survival time |  |  |  |  |  |  |
| Nesting | Intercept | 1.836 | 0.271 | 15.852 | 6.766 | **<0.001** |
|  | Mass | 0.606 | 0.132 | 28.111 | 4.515 | **<0.001** |
|  | Nesting, holes | 0.388 | 0.250 | 9.356 | 1.555 | 0.153 |
|  | Nesting, large cavity | -0.371 | 0.369 | 6.450 | -1.004 | 0.351 |
| Location | Intercept | 2.230 | 0.261 | 12.238 | 8.543 | **<0.001** |
|  | Mass | 0.599 | 0.137 | 29.34 | 4.368 | **<0.001** |
|  | Location, below ground | -0.4897 | 0.276 | 7.992 | -1.772 | 0.114 |
|  | Location, both | -0.209 | 0.269 | 6.373 | -0.778 | 0.465 |
| Sociality | Intercept | 1.507 | 0.343 | 7.034 | 4.392 | **0.003** |
|  | Mass | 0.681 | 0.144 | 47.154 | 4.724 | **<0.001** |
|  | polymorphic | 1.037 | 0.462 | 8.068 | 2.243 | 0.055 |
|  | Solitary | 0.608 | 0.351 | 6.274 | 1.730 | 0.132 |
| WLR |  |  |  |  |  |  |
| Nesting | Intercept | -0.540 | 0.162 | 28.113 | -2.774 | **<0.001** |
|  | Mass | -0.463 | 0.095 | 31.162 | -4.869 | **<0.001** |
|  | Nesting, holes | -0.178 | 0.126 | 16.471 | -1.408 | 0.178 |
|  | Nesting, large cavity | 0.272 | 0.183 | 11.800 | 1.482 | 0.165 |
| Location | Intercept | -0.563 | 0.143 | 19.475 | -3.943 | **<0.001** |
|  | Mass | -0.422 | 0.104 | 35.694 | -4.082 | **<0.001** |
|  | Location, below ground | 0.235 | 0.146 | 13.643 | 1.606 | 0.131 |
|  | Location, both | 0.121 | 0.151 | 11.619 | 0.804 | 0.438 |
| Sociality | Intercept | -0.192 | 0.166 | 10.182 | -1.154 | 0.275 |
|  | Mass | -0.471 | 0.100 | 33.167 | -4.724 | **<0.001** |
|  | polymorphic | -0.302 | 0.234 | 10.492 | -1.291 | 0.225 |
|  | Solitary | -0.402 | 0.168 | 9.213 | -2.400 | **0.039** |
| CWC |  |  |  |  |  |  |
| Nesting | Intercept | 0.486 | 0.065 | 30.743 | 7.508 | **<0.001** |
|  | Mass | 0.020 | 0.038 | 38.849 | 0.537 | 0.594 |
|  | Nesting, holes | -0.054 | 0.052 | 17.027 | -1.032 | 0.317 |
|  | Nesting, large cavity | 0.138 | 0.077 | 11.659 | 1.800 | 0.098 |
| Location | Intercept | 0.482 | 0.061 | 21.696 | 7.880 | **<0.001** |
|  | Mass | 0.009 | 0.042 | 63.493 | 0.208 | 0.836 |
|  | Location, below ground | -0.003 | 0.066 | 14.439 | -0.039 | 0.970 |
|  | Location, both | -0.070 | 0.069 | 14.003 | -1.016 | 0.327 |
| Sociality | Intercept | 0.618 | 0.068 | 11.340 | 9.035 | **<0.001** |
|  | Mass | 0.010 | 0.039 | 42.938 | 0.250 | 0.804 |
|  | polymorphic | -0.237 | 0.096 | 11.960 | -2.473 | **0.029** |
|  | Solitary | -0.170 | 0.069 | 10.171 | -2.458 | **0.033** |

**Table S4**. Number of individual bees and species represented in each nesting guild, nest location, and sociality group included in the principal component analysis. See also Figure 2.

| **Group** | **# specimens** | **# species** |
| --- | --- | --- |
| Nesting guild |  |  |
| Ground | 37 | 6 |
| Holes | 102 | 11 |
| Large cavities | 52 | 2 |
| Nest location |  |  |
| Above ground | 87 | 6 |
| Below ground | 54 | 7 |
| Both | 50 | 6 |
| Sociality |  |  |
| Eusocial | 52 | 2 |
| Socially polymorphic | 33 | 2 |
| Solitary | 106 | 15 |

**Table S5.** Results of the linear mixed-effects model predicting survival time under desiccating conditions in 15 bee species as a function of water loss rate (Log_10_WLR), critical water content (ArcCWC), and body wet mass (Log_10_Mass). In these analyses, seven species represented by a single specimen were excluded. The final best-fitting model included all fixed effects and a single interaction, as indicated below. Species and collection date were included as random effects.

| **Predictor** | **Estimate (**β**)** | **Std. Error** | **df** | **t-value** | **p-value** |
| --- | --- | --- | --- | --- | --- |
| Intercept | 3.128 | 0.167 | 146.77 | 18.69 | **<0.001** |
| Log_10_WLR | -0.957 | 0.039 | 181.08 | -24.60 | **<0.001** |
| ArcCWC | -3.373 | 0.304 | 175.92 | -11.08 | **<0.001** |
| Log_10_Mass | 0.650 | 0.115 | 131.23 | 5.64 | **<0.001** |
| ArcCWC × Log_10_Mass | -0.909 | 0.211 | 172.79 | -4.31 | **<0.001** |
|  |  |  |  |  |  |
| **Random effects** |  |  |  |  |  |
| **Group** | **Variance** | **Std. Dev.** |  |  |  |
| Species | 0.015 | 0.121 |  |  |  |
| Collection date | 0.009 | 0.093 |  |  |  |
| Residual | 0.012 | 0.109 |  |  |  |

**Table S6.** Comparison of linear mixed models assessing the influence of body mass and hair length as predictors of water loss rate and critical water content across 15 bee species. In these analyses, seven species represented by a single specimen were excluded. For each response variable, we fitted models initially including each morphological trait independently, then expanding them to include both morphological traits and their interactions. Model comparison using Akaike’s Information Criterion (AIC) was used to select the best-fitting model for each response variable. Best models and significant effects are indicated in bold (p < 0.05). Species and collection date were used as random effects.

| **Model** | **Fixed effects** | **DF** | **AIC** | **Estimate ± SE** | **T-value** | **p-value** |
| --- | --- | --- | --- | --- | --- | --- |
| Water Loss Rate | | | | | | |
| A | Mass | 5 | **26.62** | -0.454 ± 0.10 | -4.517 | **<0.001** |
| B | Hair length | 5 | 44.93 | -0.124 ± 0.09 | -1.313 | 0.198 |
| C | Mass + Hair length | 6 | 30.47 | -0.515 ± 0.12 (Mass) | -4.467 | **<0.001** |
|  |  |  |  | 0.133 ± 0.10 (Hair length) | 1.275 | 0.208 |
| D | Mass * Hair length | 7 | 27.40 | -0.208 ± 0.16 (Mass) | -1.274 | 0.209 |
|  |  |  |  | -0.167 ± 0.15 (Hair length) | -1.123 | 0.264 |
|  |  |  |  | -0.398 ± 0.14 (Interaction) | -2.814 | **0.006** |
| Critical Water Content | | | | | | |
| E | Mass | 5 | -358.4 | 0.009 ± 0.04 | 0.213 | 0.832 |
| F | Hair length | 5 | **-362.0** | 0.096 ± 0.04 | 2.763 | 0.008 |
| G | Mass + Hair length | 6 | -356.0 | -0.030 ± 0.04 (Mass) | -0.680 | 0.498 |
|  |  |  |  | 0.108 ± 0.04 (Hair length) | 2.700 | **0.008** |
| H | Mass * Hair length | 7 | -350.1 | -0.014 ± 0.06 (Mass) | -0.233 | 0.817 |
|  |  |  |  | 0.096 ± 0.06 (Hair length) | 1.67 | 0.098 |
|  |  |  |  | -0.018 ± 0.05 (Interaction) | -0.329 | 0.743 |

**Table S7**. Results of the linear mixed-effects model assessing differences in survival time under desiccating conditions, water loss rate (WLR), and critical water content (CWC) among nesting guild, nest location, and sociality across 15 species. In these analyses, seven species represented by a single specimen were excluded. This table presents the estimated coefficients, standard errors, t-values, and p-values for the fixed effects in models analyzing the response variables: Survival time, water loss rate (WLR), and critical water content (CWC). Factors such as nesting, nest location, and sociality were included in the models, with results for each factor provided.

| **Response** | **Predictor** | **Estimate (**β**)** | **S.E.** | **df** | **t-value** | **p-value** |
| --- | --- | --- | --- | --- | --- | --- |
| Survival |  |  |  |  |  |  |
| Nesting | Intercept | 2.377 | 0.367 | 10.287 | 6.475 | **<0.001** |
|  | Mass | 0.769 | 0.145 | 29.283 | 5.305 | **<0.001** |
|  | Nesting, holes | -0.039 | 0.343 | 6.854 | -0.115 | 0.912 |
|  | Nesting, large cavity | -0.802 | 0.451 | 5.575 | -1.779 | 0.129 |
| Location | Intercept | 2.351 | 0.298 | 11.451 | 7.878 | **<0.001** |
|  | Mass | 0.800 | 0.153 | 45.165 | 5.238 | **<0.001** |
|  | Location, below ground | -0.179 | 0.384 | 6.586 | -0.465 | 0.657 |
|  | Location, both | -0.084 | 0.363 | 5.322 | -0.231 | 0.826 |
| Sociality | Intercept | 1.569 | 0.311 | 5.378 | 5.045 | **0.003** |
|  | Mass | 0.803 | 0.147 | 33.264 | 5.469 | **<0.001** |
|  | polymorphic | 1.217 | 0.434 | 7.202 | 2.801 | **0.026** |
|  | Solitary | 0.667 | 0.319 | 4.297 | 2.089 | 0.100 |
| WLR |  |  |  |  |  |  |
| Nesting | Intercept | -0.560 | 0.203 | 15.607 | -2.759 | **0.014** |
|  | Mass | -0.487 | 0.101 | 22.544 | -4.816 | **<0.001** |
|  | Nesting, holes | -0.074 | 0.162 | 10.557 | -0.457 | 0.657 |
|  | Nesting, large cavity | 0.354 | 0.213 | 8.545 | 1.663 | 0.133 |
| Location | Intercept | -0.571 | 0.152 | 13.553 | -3.748 | **<0.001** |
|  | Mass | -0.446 | 0.109 | 26.112 | -4.112 | **<0.001** |
|  | Location, below ground | 0.170 | 0.177 | 8.153 | 0.962 | 0.364 |
|  | Location, both | 0.088 | 0.171 | 6.677 | 0.515 | 0.623 |
| Sociality | Intercept | -0.204 | 0.162 | 7.400 | -1.262 | 0.245 |
|  | Mass | -0.480 | 0.100 | 22.856 | -4.775 | **<0.001** |
|  | polymorphic | -0.303 | 0.233 | 8.973 | -1.301 | 0.226 |
|  | Solitary | -0.427 | 0.165 | 6.398 | -2.582 | **0.039** |
| CWC |  |  |  |  |  |  |
| Nesting | Intercept | 0.391 | 0.083 | 17.689 | 4.696 | **<0.001** |
|  | Mass | -0.005 | 0.040 | 34.965 | -0.124 | 0.902 |
|  | Nesting, holes | 0.019 | 0.068 | 10.612 | 0.278 | 0.787 |
|  | Nesting, large cavity | 0.218 | 0.092 | 8.960 | 2.372 | **0.042** |
| Location | Intercept | 0.463 | 0.067 | 15.878 | 6.924 | **<0.001** |
|  | Mass | -0.009 | 0.044 | 61.725 | -0.209 | 0.836 |
|  | Location, below ground | -0.050 | 0.083 | 9.331 | -0.606 | 0.559 |
|  | Location, both | -0.070 | 0.083 | 9.040 | -0.844 | 0.420 |
| Sociality | Intercept | 0.609 | 0.069 | 7.940 | 8.803 | **<0.001** |
|  | Mass | -0.007 | 0.040 | 31.437 | -0.181 | 0.857 |
|  | polymorphic | -0.254 | 0.098 | 9.352 | -2.593 | **0.028** |
|  | Solitary | -0.194 | 0.071 | 6.891 | -2.719 | **0.030** |
